# Complete mitochondrial genomes of biological control stains in the *Trichoderma harzianum* Rifai complex (strains DL1-3, KC1-1, and PAR10) isolated from Californian grapevines

**DOI:** 10.1080/23802359.2025.2552822

**Published:** 2025-09-02

**Authors:** Christopher M. Wallis, Jianchi Chen

**Affiliations:** Crop Diseases, Pests and Genetics Research Unit, U.S. Department of Agriculture-Agricultural Research Service, San Joaquin Valley Agricultural Sciences Center, Parlier, CA, USA

**Keywords:** Biocontrol fungi, grapevine, fungal trunk disease, Pierce’s disease, *Xylella fastidiosa*

## Abstract

The complete mitochondrial genomes of *Trichoderma* sp. strains DL1-3 (29,197 bp), KC1-1 (27,631 bp), and PAR10 (29,122 bp), isolated from grapevine leaves, were obtained. All strains contained 25 or 26 tRNA genes, two rRNA genes, and 15 core mitochondrial protein coding genes. Phylogenetic trees made with housekeeping region and mitochondrial sequences placed these strains in the *T. harzianum* clade, with DL1-3 and PAR10 as potential novel species, and KC1-1 identified as a strain of *T. harzianum*. This suggests that complete mitochondria genome comparisons provide valuable information in resolving questions of species identification, including putatively identifying those that are novel.

## Introduction

Grapevines and fruiting trees encounter a wide variety of fungal trunk diseases that limit yields (Gams 2000; Gramaje et al. 2018; Crous and Travadon et al. 2022). Long-term control may involve biopesticides (Baumgartner et al. 2019), including using various species in the *Trichoderma harzianum* Rifai 1969 complex. Thus, *Trichoderma* sp. strains were acquired from Californian vineyards and screened for ability to control diseases. Of these, three strains, DL1-3, KC1-1, and PAR10, exhibited good potential to limit pathogen growth (Supplementary Figure S1).

The systematics of *Trichoderma* species remain in flux, with the genera being continually revised (Samuels et al. 2012; Samuels and Hebbar 2015). Key to allowing this taxon to be accurately resolved is further understanding of its genetics (Chaverri et al. 2015), which could include obtaining and comparing mitochondrial genomes amongst strains. Thus, the *Trichoderma* strains DL1-3, KC1-1, and PAR10 underwent next-generation sequencing to obtain their mitochondrial genomes for comparison with those previously published. Results could be a useful component in structuring the *Trichoderma* taxon.

## Materials and methods

*Trichoderma* strains were isolated in August 2022 from grapevine leaves collected from Delano, CA (35°46′30″N, 119°15′02″W) for DL1-3, King City, CA (36°13′06″N, 121°08′23″W) for KC1-1, or Parlier, CA (36°35′33″N, 119°31′11″W) for PAR10 in August 2022. Strains were isolated from grapevine leaves from those locations and obtained via tip culturing. All strains were maintained on potato dextrose agar (BD Difco, Franklin Lakes, NJ) and were identified as belonging to the *Trichoderma harzianum* complex based on culture, conidia and mycelia branching pattern morphology (Samuels and Hebbar 2015). Phylogenetic trees derived from housekeeping *its*, *rpb2*, and *tef1* genes sequences, obtained as described below, further resolved species (Supplementary Figure S2). Strains were deposited in the U.S. Department of Agriculture-Agricultural Research Service Culture Collection in Peoria, IL (https://nrrl.ncaur.usda.gov/; contact: NRRL curator, NRRLcollectionmanager@usda.gov), with accession number NRRL#64840 for DL1-3, NRRL#64841 for KC1-1, and NRRL#64839 for PAR10.

DNA was extracted via the NucleoSpin Plant II mini kit (Macherey-Nagel, Allentown, PA) following instructions for fungal material. DNA quality was accessed using Qubit (Thermo-Fisher Scientific, Waltham, MA) and a Tape Station (Agilent, Santa Clara, CA) following manufacturer’s protocols. Sequencing was performed using an NovaSeq 6000 PE150 platform (Illumina, San Diego, CA) by a contract service (Novogene, Davis, CA) yielding 33.4 GB of data for DL1-3, 36.84 GB for KC1-1, and 39.88 GB for PAR10. For each of the three strains, reads were assembled using the SPAdes (Ver. 3.14.0) assembler (Bankevich et al. 2012). Assembly quality was assessed using QUAST (ver. 5.2.0) (Gurevich et al. 2013). Tablet (Milne et al. 2013) was used to visualize the resultant SPAdes fastg file to identify the circular contigs of from each strain that were potentially the mitochondrial genomes. Stand-alone BLAST+ (ver. 2.7.1) (Camacho et al. 2009) of these circular contigs against the mitochondrial genome for *Trichoderma harzianum* (GenBank accession MN564945.1) verified the mitochondrial genomes. Overlap regions on the 5′ and 3′ ends were detected by self-BLAST analysis (BLAST+) and were trimmed manually (Wallis et al. 2022). Nucleotide coverage of each mitogenome was estimated by BWA mapping (Li and Durbin 2010) with 15,032 for DL1-3, 4901 for KC1-1, and 13,465 for PAR10 (Supplementary Figure S3).

Mitochondrial genome was annotated using the online tool GeSeq (ver. 2.03) (Tillich et al. 2017) in which coding sequences were identified using the BLAT search module (ver. 35) and tRNA sequences identified by BLAT, ARAGORN (ver. 1.2.38), and ARWEN (ver. 1.2.3). Visualization of the mitochondrial genomes was made using Proksee Map Builder version 1.4.4 (Grant et al. 2023).

A phylogenetic analysis was performed using the mitochondrial genomes of DL1-3, KC1-1, and PAR10 along with those of *Trichoderma* species and *Fusarium globosum* (as an outgroup) available on GenBank. The software MEGA (version 11.0.13) was utilized to perform the required analyses (Tamura et al. 2021). First, nucleotide sequences were aligned using MUSCLE (Edgar 2021) with default settings. Next, the coding sequences, with introns removed, were combined into a final alignment file. The alignment file was then used to make a maximum-likelihood tree in MEGA (version 11.0.13) (Tamura et al. 2021) with the Tamura–Nei model and bootstrapping with 500 replications.

## Results and discussion

**Figure 1.**
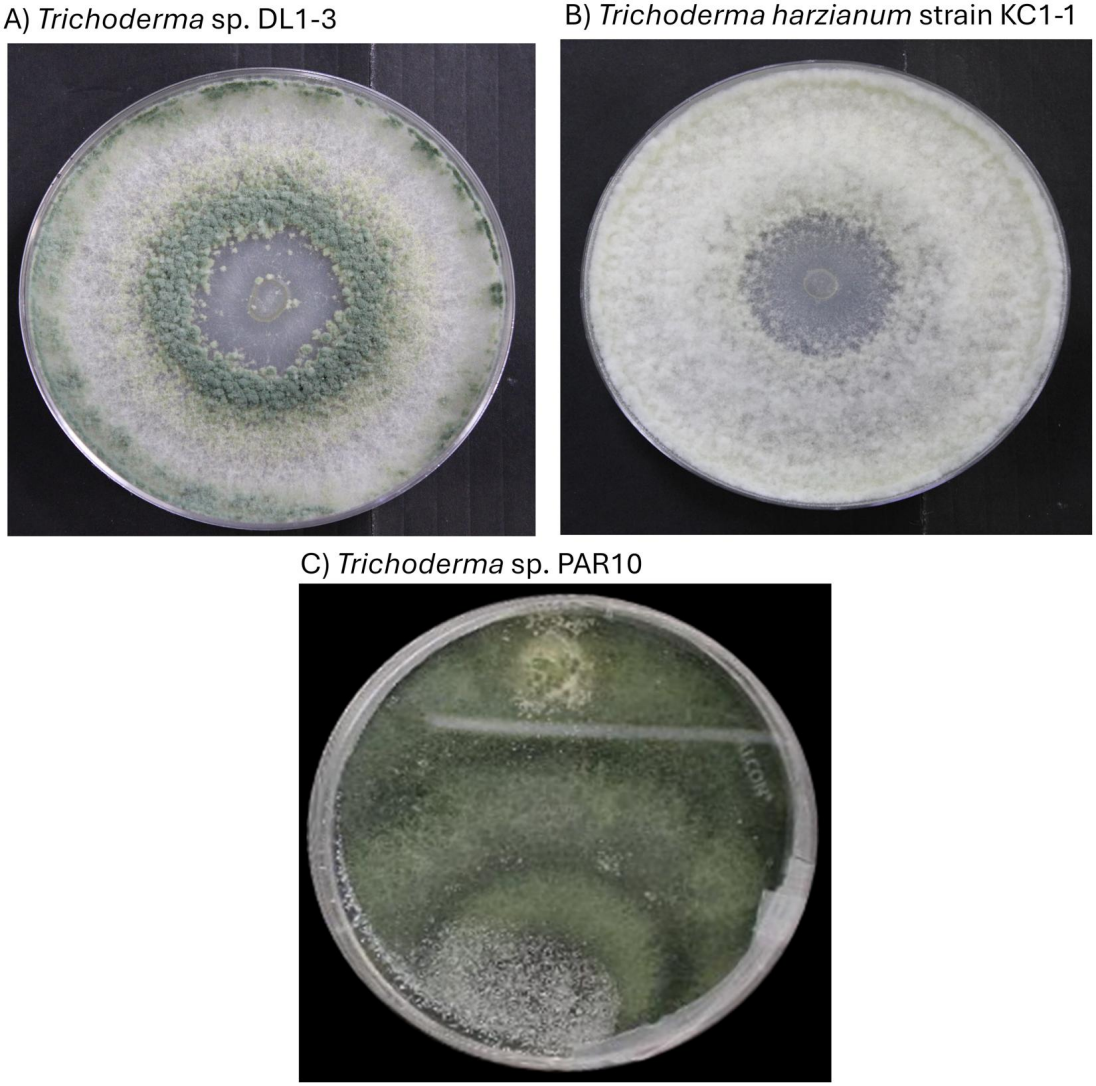
Author-provided photographs of one-week-old cultures of *Trichoderma* sp. strains. (A) DL1-3, (B) KC1-1, and (C) PAR10. Note the green spore production characteristic of the *Trichoderma* genus, and differences in the patterns of spore production between the strains. The green spores were the densest on cultures of *T. harzianum* strain KC1-1, and the most widespread on *T.* sp. strain PAR10.

All three strains exhibit growth and morphological characters similar to *T. harzianum* (Figure 1). Likewise, phylogenetic trees using the curated sequences of the *its*, *rpb2*, and *tef1* regions pace DL1-3, KC1-1, and PAR10 into the *T. harzianum* complex, albeit different species within the complex (Cai and Druzhinina 2021) (Supplementary Figure 2).

**Figure 2.**
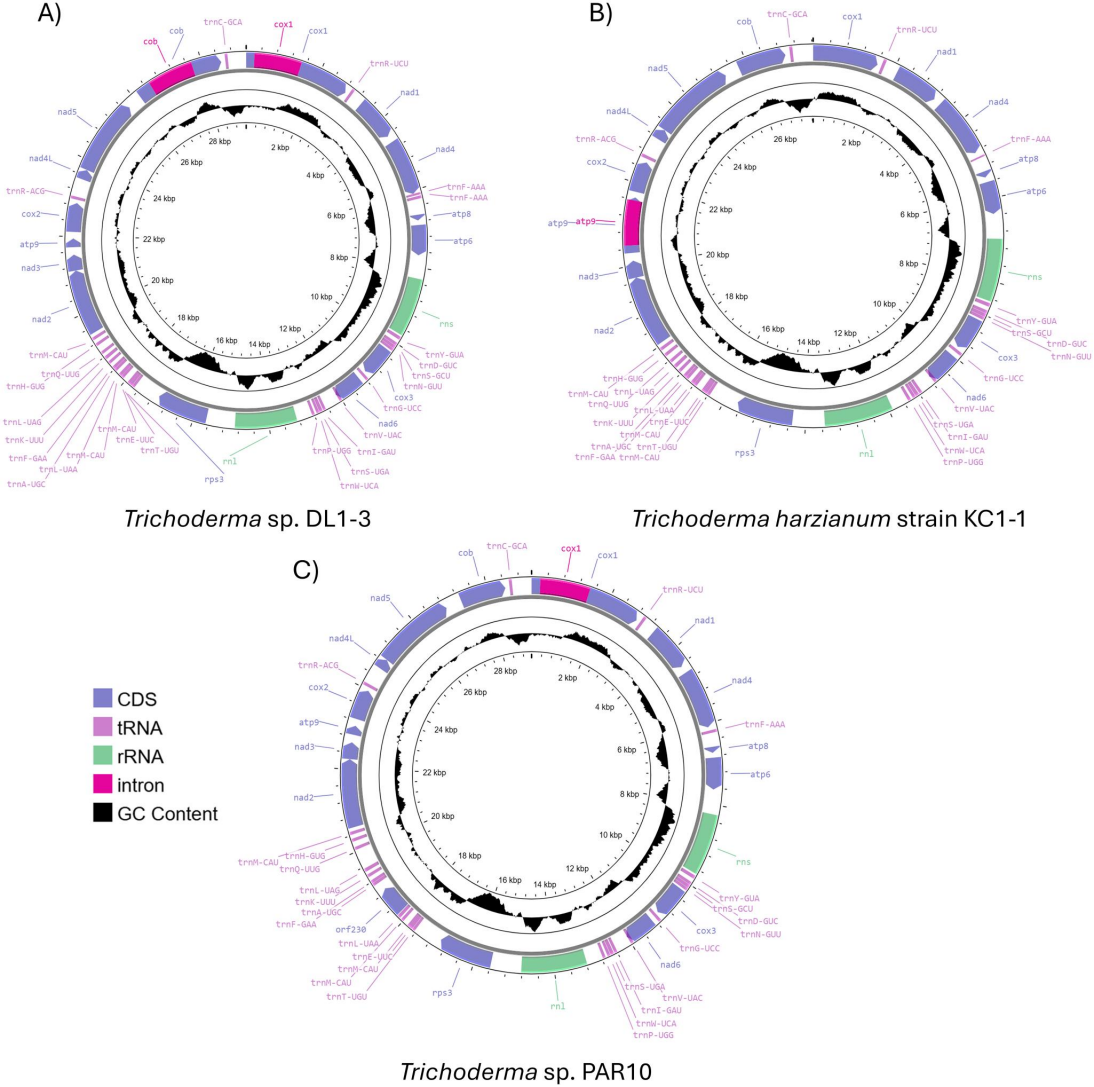
Annotation maps of the mitochondrial genomes of *Trichoderma* sp. strains. (A) DL1-3, (B) KC1-1, and (C) PAR10. All genes were present on the heavy strand, and GC content was shown in the middle circle.

The final completed, circular mitochondrial genomes is 29,197 bp for DL1-3, 27,631 bp for KC1-1, and 29,122 bp for PAR10 (Figure 2). GC content is 27.45% for DL1-3, 27.55% for KC1-1, and 27.57% for PAR10.

Mitochondrial genome annotations reveal that all three strains have genes for three ATP synthase subunits, one gene for cytochrome b, three genes for cytochrome c oxidase subunits, seven genes for NADH dehydrogenase subunits, 25 (KC1-1 and PAR10) or 26 (DL1-3) genes coding for transfer RNAs, two genes coding for ribosomal RNAs, and a gene coding for ribosomal protein S3 (Figure 2). PAR10 has an additional open reading frame (*orf230*). All genes are on the heavy strand. DL1-3 and PAR10 have a 1246 bp intron in the cytochrome c oxidase subunit 1 gene at the nucleotide position 219. DL1-3 also have a 1206 bp intron in the cytochrome b gene at the nucleotide position 394. KC1-1 has a 1080 bp intron in the ATP synthase subunit 9 gene at the nucleotide position 182.

**Figure 3.**
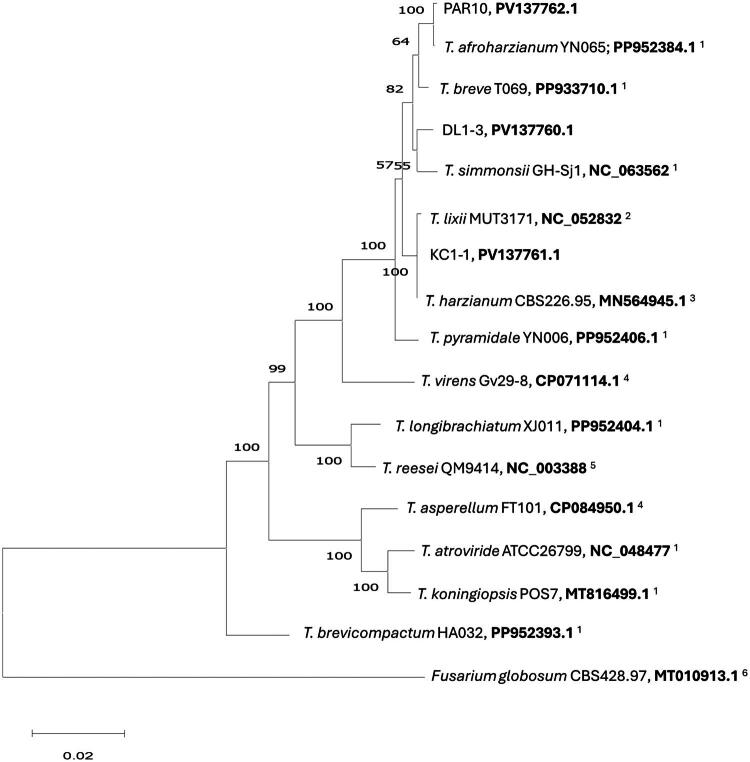
Phylogenetic tree of coding regions of the 12 proteins and two rRNA sequences found present in *Trichoderma* species mitochondrial genomes, with intron regions removed and *Fusarium globosum* as the outgroup (with branch trimmed for clarity). The maximum-likelihood method was used with bootstrap value above each branch and based on 500 iterations and converted to %. Strain name and GenBank Accession numbers are provided. The following sequences were used: *Fusarium globosum* MT010913.1 (Brankovics et al. 2020), *Trichoderma* sp. PAR10 PV137762.1 (this study), *Trichoderma afroharzianum* PP952384.1 (unpublished), *Trichoderma breve* PP933710.1 (unpublished), *Trichoderma* sp. DL1-3 PV137760.1 (this study), *Trichoderma simmonsii* MZ292901.1 (unpublished), *Trichoderma lixii* MT495248.1 (Venice et al. 2020), *Trichoderma harzianum* strain KC1-1 (this study), *Trichoderma harzianum* MN56945.1 (Kwak 2021), *Trichoderma pyramidale* PP952406.1 (unpublished), *Trichoderma virens* CP071114.1 (Li et al. 2021), *Trichoderma longibrachiatum* PP952404.1 (unpublished), *Trichoderma reesei* AF447590.1 (Chambergo et al. 2002), *Trichoderma asperellum* CP084950.1 (Li et al. 2021), *Trichoderma atroviride* MN125601.1 (unpublished), *Trichoderma koningiopsis* MT816499.1 (unpublished), and *Trichoderma brevicompactum* PP952393.1 (unpublished). The bar represents the distance 0.02.

Phylogenetic analysis clusters the three strains (DL1-3, KC1-1, and PAR10) along with several related species in the *T. harzianum* complex (Figure 3). Identification guidelines based on housekeeping genes (Cai and Druzhinina 2021) suggest DL1-3 as a novel species. PAR10 also is concluded as a putative novel species based on housekeeping genes, and herein it appears one with close homogeny to *T. afroharzianum*. However, KC1-1 groups with *T. harzianum* according to the mitochondrial genome-derived phylogenetic tree, which is confirmed with conclusions based on housekeeping genes that verified this is a strain of *T. harzianum*.

This work provides three addition mitochondrial genomes for the genus *Trichoderma*, with strains obtained from a geographical region that is not well represented (western North America) as only one mitochondrial genome from a *Trichoderma* from this region has been published (Wallis et al. 2022). Although *Trichoderma* as a genus has strains used for a multitude of industrial and agricultural purposes, the lack of genetic information hampers species descriptions and understanding of ecological and taxonomic differences, both within species and among species (Gorman et al. 2023). These results, although demonstrating not large differences in the number of introns, tRNAs, or coding regions, do show some diversity in mitochondrial genomes.

## Conclusions

The study obtained mitochondrial genomes of *Trichoderma* species strains DL1-3, KC1-1, and PAR10, which were selected for biological control potential to manage grapevine diseases. Phylogenetic analyses highlighted that these strains were related species in the *T. harzianum* complex, with KC1-1 a *T. harzianum* strain and DL1-3 and PAR10 as putative novel species, which was consistent with conclusions reached by more traditional approaches using *rpb2* and *tef1* regions (Cai and Druzhinina 2021). Thus, these mitochondrial genomes improved understanding about *Trichoderma* strains and could shed light on the diversity of these fungi.

## Supplementary Material

Supplementary Data.pdf

## Data Availability

The data that support the findings of this study are available in the National Center for Biotechnology Information GenBank (NCBI-GenBank) at https://www.ncbi.nlm.nih.gov/, reference number (BioProject Number) PRJNA1101532. These data were derived from the following resources available in the public domain: for DL1-3, BioSample SAMN41062247, SRA SRR32684203, and GenBank Accession number is PV137760; for KC1-1 BioSample SAMN41062245, SRA SRR32684204, and GenBank Accession PV137761; and for PAR10 BioSample SAMN41062124, SRA SRR32671381, and GenBank Accession PV137762.
