# Supplementary material for: Complete mitochondrial genomes of biological control stains in the Trichoderma harzianum Rifai complex (strains DL1-3, KC1-1, and PAR10) isolated from Californian grapevines
Source: Mitochondrial DNA B Resour. 2025 Sep 2;10(10):893–8. doi: 10.1080/23802359.2025.2552822 (PMC12406320; doi:10.1080/23802359.2025.2552822)
Supplement: Supplementary Data.pdf [file TMDN_A_2552822_SM2361.pdf]

Supplementary Data for:
Complete mitochondrial genomes of biological control stains in the *Trichoderma harzianum* Rifai complex (strains DL1-3, KC1-1, and PAR10) isolated from Californian grapevines

Christopher M. Wallis*, Jianchi Chen

Crop Diseases, Pests and Genetics Research Unit, U.S. Department of Agriculture- Agricultural Research Service, San Joaquin Valley Agricultural Sciences Center, Parlier, CA 93648, U.S.A.

ORCID: C. M. Wallis 0000-0003-3855-8256; J. Chen 0000-0003-0662-3164

* Corresponding author: C. M. Wallis; E-mail: christopher.wallis@ars.usda.gov

Keywords: biocontrol fungi, grapevine, fungal trunk disease, Pierce's disease, *Xylella fastidiosa*

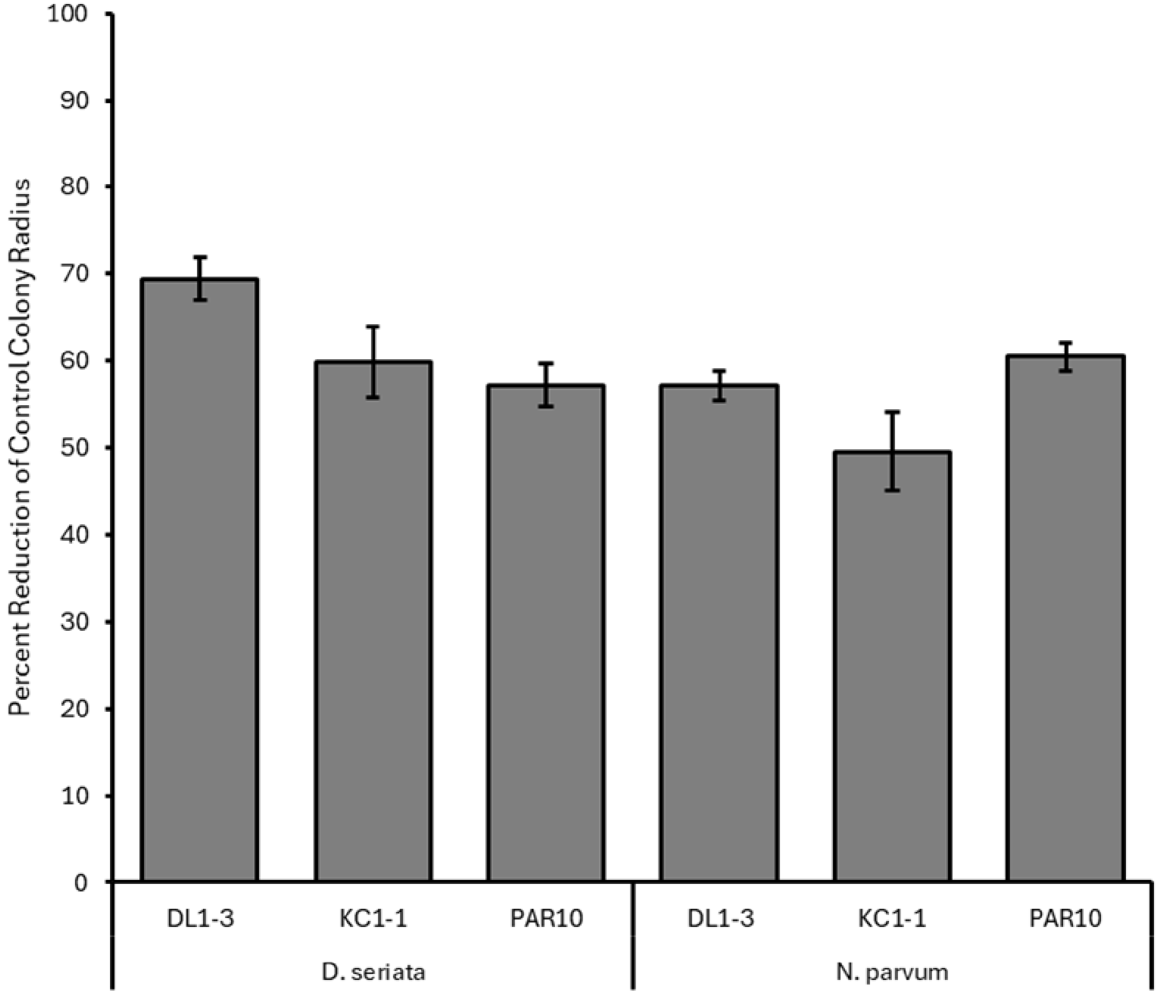

Figure S1. Percent of colony reduction of two grapevine trunk fungal pathogens (*Diplodia seriata* and *Neofusicoccum parvum*) when co-plated with *Trichoderma* species strains DL1-3, KC1-1, or PAR10.

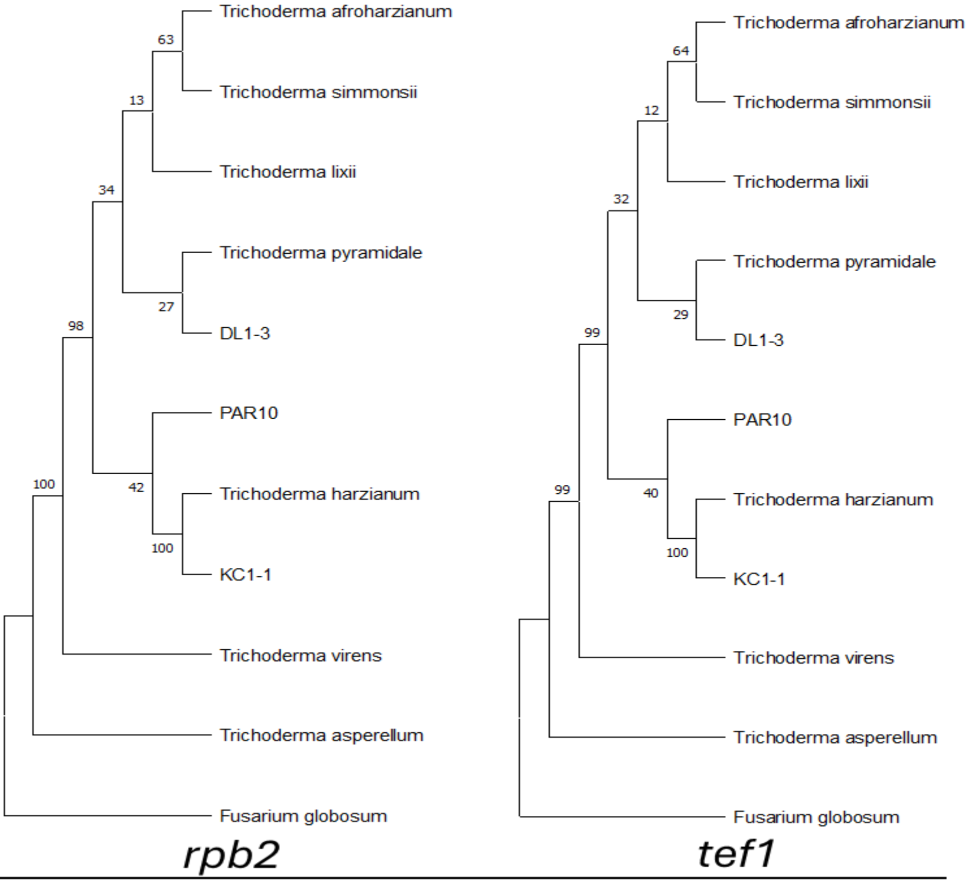

Species	*rpb2*	*tef1*
Fusarium globosum	OK000552.1	MT010993.1
Trichoderma afroharzianum	PQ559192.1	OQ509672.1
Trichoderma asperellum	EU248617.1	MT681982.1
Trichoderma harzianum	AF545549.1	OP970249.1
Trichoderma lixii	KJ665290.1	MT435114.1
Trichoderma pyramidale	KJ665334.1	KJ665699.1
Trichoderma simmonsii	OP714168.1	OQ116869.1
Trichoderma virens	PQ559194.1	OQ858777.1

Figure S2. Phylogenetic trees of housekeeping genetic regions *rpb2* and *tef1* for *Trichoderma* species strains DL1-3, KC1-1, and PAR10 with 13 other Trichoderma species sequences obtained from the NCBI GenBank database. Accession numbers are provided in the Table below the trees. Trees were made using MEGA (Tamura et al. 2021) using a Maximum Likelihood Tree with bootstrapping performed (500 iterations). Species identifications were made using a criterion of over 99% identity of *rpb2* with a known species, and over 96% identity of *tef1* with a known species (Cai and Druzhinina 2021). Based on this, DL1-3 and PAR were concluded to be novel species (the criteria were not met), but KC1-1 was confirmed to be a strain of *T. harzianum.*

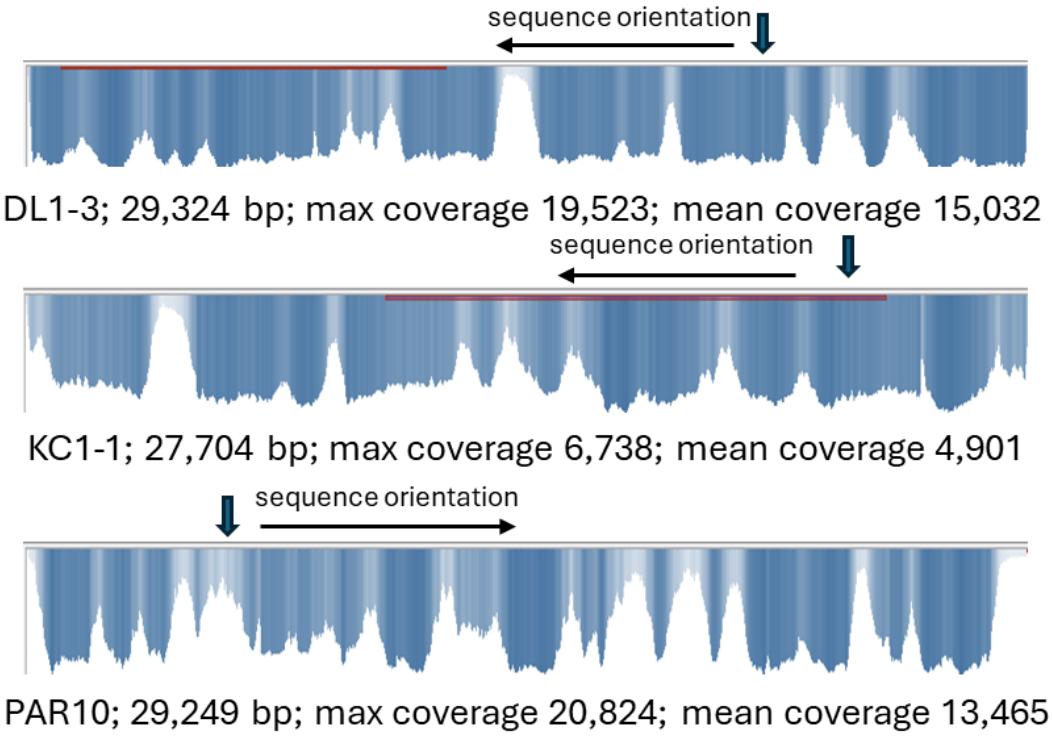

Figure S3. Coverage maps for the contig containing the mitochondrial genome for *Trichoderma* strains DL1-3, KC1-1, and PAR10. Note these coverage maps represent the non-circularized contigs and were not trimmed, and final DL1-3 and KC1-1 were reverse complimented to match existing sequences in GenBank. The arrows represent the first nucleotide in the final published mitochondrial genomes. Coverage statistics are provided. Low coverage regions had at least 200 nucleotide coverage as a minimum and generally contained A-T rich sequences.
